# Effects of polymorphism of the *GPAM* gene on milk quality traits and its relation to triglyceride metabolism in bovine mammary epithelial cells of dairy cattle

**DOI:** 10.5194/aab-64-35-2021

**Published:** 2021-01-29

**Authors:** Haibin Yu, Yaolu Zhao, Ambreen Iqbal, Lixin Xia, Zitong Bai, Hao Sun, Xibi Fang, Runjun Yang, Zhihui Zhao

**Affiliations:** 1 College of Animal Science, Jilin University, Changchun, Jilin 130062, PR China; 2 Institute of Muscle Biology and Growth, Leibniz Institute for Farm Animal Biology (FBN), 18196 Dummerstorf, Germany; 3 College of Coastal Agricultural Sciences, Guangdong Ocean University, Zhanjiang, Guangdong 524088, PR China

## Abstract

Mitochondrial glycerol-3-phosphate acyltransferase (*GPAM*) catalyses the initial and rate-regulated first-stage pathway of glycerol lipid synthesis and helps to allocate acyl-CoA (acyl-coenzyme A) to triglyceride (TG) synthesis and away from degradation pathways in animal lipometabolism-related pathways. In this study, RNA interference (RNAi) and *GPAM* gene overexpression were used to examine the correlation between the expression of *GPAM* and adipogenesis in bovine mammary epithelial cells (bMECs). Additionally, three novel polymorphisms were identified within the bovine key functional domain of *GPAM* with Sanger sequencing. The relationship between variants of the *GPAM* gene and milk quality traits of Chinese Holstein cows was then analysed using statistical methods. The results showed that knockdown of the *GPAM* gene significantly reduced the synthesis of triglycerides in the bMECs (p < 0.05), whereas the overexpression of the *GPAM* gene significantly increased the synthesis of TG (p < 0.05). In Chinese Holstein dairy cattle, the polymorphic locus of the *GPAM* gene E20-3386G > A was significantly correlated with fat, protein and somatic cell count (p < 0.05); I18-652A > G was significantly correlated with fat, total fat content, protein, dry matter and somatic cell count (p < 0.05); and I18-726A > G was significantly correlated with protein, milk yield, dry matter and somatic cell count (p < 0.05). Specifically, individuals with the AA genotype of the I18-652A > G and E20-3386G > A polymorphic loci had a higher milk fat percentage (p < 0.05). In summary, *GPAM* plays a pivotal role in the intracellular regulation of triglyceride, and its mutations could work as a competent molecular marker for selective breeding in dairy cattle.

## 1 Introduction

Milk is defined as the secretion from a mammal's mammary glands, and the key function of milk is to provide nutrition to mammalian young. The milk of some animals like buffalos, cows, sheep and goats is also used for human consumption. Worldwide, dairy farms produced about $820\times 10^{6}$ tons of milk in 2016 (Salter, 2017). According to an estimation from the Food and Agriculture Organization (FAO), 85 % of all of the milk produced worldwide in 2011 came from cows (Gerosa and Skoet, 2012). Due to a change in consumer demand and preference towards milk fat, which is considered to be a good source of dietary fat, milk fat is currently used as an indicator of milk quality. This has prompted livestock farmers to produce dairy products with a diverse range of fat contents, thereby urging researchers to explore the molecular mechanisms governing milk fat production (Shen et al., 2016). Milk quality for the farmer is related to the price, but the price of milk depends on fat, protein content and somatic cell count. There are many factors that affect milk quality, with the most important of these being genetics, nutrition and the management of animals, the lactation amount and the stages of lactation (Vikram and Jancy, 2016). The major constituent of milk fat is triglycerides (TGs), which comprise almost 97 % to 98 % of the milk fat content. Milk fat also includes fatty acids (FA) and cholesterol (CHO) as minor components (Jiang et al., 2020). TGs are an important part of milk, and their synthesis in mammary cells of cattle can affect the milk fat percentage (Holub and Kuksis, 1972).

*GPAM* belongs to the *GPAT* gene family and is located at chromosome 26q22 in bovine. The *GPAM* gene is also called *GPTA1* and *mtGPAT*. The gene sequence of *GPAM* shows that it contains 20 exons that encode for protein (Roy et al., 2002, 2006). The *GPAM* gene catalyses the first committed steps of phospholipid and triglyceride (TG) biosynthesis (Roy et al., 2006). Previous work has proven that the *GPAM* gene plays a dynamic role in triglyceride (TG) synthesis, such as the 10-fold increase in the activity of the *GPAM* gene in 3T3-L1 adipocyte differentiation (Coleman et al., 1978) and the 5-fold increase in the activity of the *GPAM* gene during hepatocyte TG synthesis in the neonatal liver (Coleman and Haynes, 1983). It has also been reported that the *GPAM* in fat storage initiates 50 % of the TGs in the adipose and liver tissue in mice (Hammond et al., 2002). Moreover, studies have reported that TG and glycerophospholipid enzyme synthesis is present at the location of *GPAM* on the outer membrane of mitochondria, which is responsible for the transportation of the lysophosphatidate products to the endoplasmic reticulum (Bell and Coleman, 1980).

Triglyceride (TG) accumulates in the body of animals due to factors such as a carbohydrate-rich diet, eating more frequently and the availability of more energy. The majority of research on TG synthesis has concentrated on the transcriptional control of genes by *PPAR* and sterol *SREBP*, although some studies have also proven that *GPAM* may regulate the synthesis of triglyceride acutely (Lewin et al., 2001). Due to advancements in technology, examination of the role of the *GPAM* gene in lipid metabolism and fat deposition in Chinese Holstein cows is now possible.

The objective of this study is to explore the regulatory role of the *GPAM* gene on adipogenesis by RNA interference (RNAi) and gene overexpression as well as to investigate single nucleotide polymorphisms of the *GPAM* gene in Chinese Holstein cows in order to identify the relationship between genotypes and milk quality traits.

## 2 Materials and methods

### 2.1 Ethics statements

All animal experiments in the present study strictly comply with the relevant regulations regarding the care and use of experimental animals issued by the Animal Protection and Use Committee of Jilin University (permit no. SYXK(Ji)pzpx20181227083).

### 2.2 Experimental materials

#### 2.2.1 Animal cell line

The mammary epithelial cells were preserved by the Laboratory of Molecular Genetics, College of Animal Science, at Jilin University.

#### 2.2.2 Animals

This study involved 241 Chinese Holstein dairy cattle from a dairy farm in Heilongjiang Province. The cows used were all from the same farm and the same batch, and their genetic backgrounds were similar. Milk was collected after the cow's second pregnancy in order to measure the related milk quality traits.

### 2.3 Traits analysis

Milk samples were collected 11 times at an interval of 30 d. Following sample collection, milk quality tests (milk yield, fat, protein, lactose, dry matter and urea) and data collection were carried out with a milk composition analyser (FOSS, MilkoScan FT3, Denmark).

### 2.4 Primers design and polymerase chain reaction (PCR) amplification

Premier 5 software was utilised to design the shRNA (short hairpin RNA) primers, the *GPAM* gene coding sequence (CDS) primers and the SNP (single nucleotide polymorphism) primers, based on the bovine *GPAM* gene's existing published sequences (ENSBTAG00000011917). All of the primers used in this study were synthesised by Genewiz (SuZhou, China). The primers' sequences are given in Table 1.

**Table 1:** Primer information used in the experiment.

Primer		Forward sequences	Reverse sequences	Target sequence	Product	Temperature
shRNA of *GPAM*	–	5${}^{\prime }$-CACCGGGTGCTGCTGAAACTG TTCATTCAAGAGATGAACAGTTT CAGCAGCACCCTTTTTTG-3${}^{\prime }$	5${}^{\prime }$-GATCCAAAAAAGGGTGCTGCT GAAACTGTTCATCTCTTGAATGA ACAGTTTCAGCAGCACCC-3${}^{\prime }$	GGGTGCTGCTGAA ACTGTTCA	–	–
Polymorphism	E20-3386G > A, I18-652A > G	5${}^{\prime }$-GTCCAACATTCCCGAGTGC-3${}^{\prime }$	5${}^{\prime }$-GCCAGATGCCAAGTCTCAAGT TCCT-3${}^{\prime }$	–	380 bp	60 ${}^{\circ }$C
	and I18-726A > G	5${}^{\prime }$-GTCCAACATTCCCGAGTGCT-3${}^{\prime }$	5${}^{\prime }$-CCGTGAAGGGTCTGCTCTTT-3${}^{\prime }$		665 bp	60 ${}^{\circ }$C
Coding region of *GPAM*	–	5${}^{\prime }$-ggatccATGGATGAATCTGCATT GACCCT-3${}^{\prime }$	5${}^{\prime }$-gcggccgcCTACAGCACCACCAA ACTCAGAATAT-3${}^{\prime }$	–	2478 bp	62 ${}^{\circ }$C
Real-time qPCR	–	5${}^{\prime }$-ATCGCATTATTAGAGGGTCA TTAC-3${}^{\prime }$	5${}^{\prime }$-CAAAGAAAGTAGGAGCAGAA ACAG-3${}^{\prime }$	–	89 bp	60 ${}^{\circ }$C

For polymerase chain reaction (PCR) amplification, a total 20 µL volume of 10 pmol L${}^{-1}$ of each primer, 140 ng of bovine genomic DNA, 5 µL of dNTP mix, 2 µL of buffer and 1.5 µL of Taq DNA polymerase (Takara), was made. The PCR amplification conditions were as follows: incubation of the PCR mixture at 95 ${}^{\circ }$C for 5 min, 35 cycles of 95 ${}^{\circ }$C for 30 s, the annealing temperature of each fragment for 30 s, 62 ${}^{\circ }$C for 1000 bp min${}^{-1}$ and a final extension at 72 ${}^{\circ }$C for 10 min.

A total volume of 50 µL was made for *GPAM* PCR amplification's open reading frame (ORF) and consisted of the following contents: 250 ng of bovine cDNA, 10 pmol L${}^{-1}$ of each primer, 10 µL of dNTP, 5 µL of buffer and 4 µL of PrimeSTAR HS DNA polymerase (Takara). The PCR amplification conditions were as follows: PCR mixture was first incubated at 95 ${}^{\circ }$C for 5 min, followed by 30 cycles of 98 ${}^{\circ }$C for 30 s, annealing temperature of each fragments for 30 s, and 72 ${}^{\circ }$C for 2 min 30 s with a final extension step at 72 ${}^{\circ }$C for 10 min.

### 2.5 Construction of the pBI-CMV3-GPAM and pGPU6-shRNA-GPAM vectors

#### 2.5.1 Target site selection for small interfering RNA (siRNA) and the design of the hairpin siRNA template oligonucleotide

The target sequence of *GPAM* is GGGTGCTGCTGAAACTGTTCA. A hairpin-like oligonucleotide sequence derived from *GPAM* mRNA target sequences and an interfering RNA were synthesised by GenePharma Corporation (Shanghai, China). The oligonucleotide sequences for the pGPU6/GFP/Neo vector are shown in Table 1.

#### 2.5.2 Construction of the pBI-CMV3-GPAM vector

The CDS sequence of *GPAM* with Not I and BamH I restriction sites was obtained using PCR. The pBI-CMV3 vector was linearised with Not I and BamH I restriction enzymes (New England Biolabs, Ipswich, MA, USA), and *GPAM*'s CDS sequence was then cloned into the linearised pBI-CMV3 plasmid by T4 ligase (Thermo Scientific, Massachusetts, USA).

### 2.6 Mammary epithelial cell culture and transfection

The bMECs were proliferated for 24 h in six-well plates (Thermo Fisher Scientific, Massachusetts, USA), and the final concentration of cells was 1.2 × 10${}^{6}$ cells per well. Each well was supplemented with 10 % fetal bovine serum (FBS; Tian Hang, Zhe Jiang, China) and incubated at 37 ${}^{\circ }$C in a 5 % CO${}_{2}$ incubator (Thermo Fisher Scientific, Massachusetts, USA).

For transfection, pBI-CMV3-GPAM and pGPU6-shRNA-GPAM vector DNA (3.0–3.5 µg) and 7.5 µL FuGENE HD transfection reagent (Promega, Madison, Wisconsin, USA) were diluted in 150 µL Opti-MEM serum-free media (Sigma-Aldrich, St. Louis, Missouri, USA) and were mixed lightly. The mixture was incubated at room temperature for 15 min and then added to the pores of each six-well plate. After 36 h of transfection, green fluorescent protein (GFP) expression was detected using a fluorescence microscope (NikonTE2000, Tokyo, Japan).

### 2.7 Detection and analysis of protein and mRNA levels of *GPAM* in bMECs

Whole RNA was extracted from bMECs with TRIzol reagent (Thermo Fisher Scientific, Massachusetts, USA). The PrimeScript™ RT reagent kit (TaKaRa, Bejing, China) was used for cDNA synthesis. Real-time qPCR (RT qPCR) was followed through with SYBR Green Real-Time PCR Master Mix (TaKaRa) utilising the specific primers shown in Table 1. In order to determine the mRNA level of *GPAM*, the threshold of the *GPAM* gene was normalised to an internal reference gene (β-*actin*) using the formula 2${}^{\text{-CT}}$ as the relative expression rate; the relative expression amount of each group was then calculated.

For the extraction of total protein, RIPA lysis buffer (Takara) was used when cell confluence reached 90 %. The cells were incubated on ice for 5 min and then whirled for 5 min; this procedure was repeated five times. Finally, the lysate was removed by centrifugation at 4 ${}^{\circ }$C with 15 000 × g for 10 min. Using SDS-PAGE (sodium dodecyl sulfate–polyacrylamide gel electrophoresis), total protein (40 µg per sample) was loaded, made up by vertical electrophoresis and transfer onto polyvinylidene fluoride or polyvinylidene difluoride (PVDF; 0.22 µm) membranes (Millipore, Massachusetts, USA). Western blotting was then performed using the following antibodies: anti-GPAM (Abcam, Cambridge, UK) and anti-β-actin (Abcam).

### 2.8 Determination of the TG content in bMECs

In serum-free DMEM/F12 medium, 1.2 × 10${}^{6}$ cells were inoculated into each well. At a total of 46 h of post-transfection, the cells were assembled at a concentration equal between control cells and positive. A triglyceride detection kit (Solarbio, Beijing, China) was utilised to evaluate the total TG content in bMECs of the *GPAM* gene that were silenced and overexpressed. At the same time, the optical density (OD) value was detected using a microplate reader (SpectraMax iD5, California, USA). The total protein concentration was then detected using an Enhanced BCA Protein Quantitation Assay Kit (KeyGEN BioTECH, Jiangsu, China).

### 2.9 SNPs detection in the *GPAM* gene and genotyping

DNA samples of 241 dairy cows were amplified by PCR. The PCR products were sequenced by Genewiz (Suzhou, China). The polymorphisms of the *GPAM* gene's key functional regions were identified by sequencing. The primers' digestion and sequences of each reaction system of SNP are given in Table 1.

### 2.10 Correlation analysis

The allelic and genotypic frequencies were worked out for the population of Chinese Holstein cows in this study. An analysis of the *GPAM* gene's genotypic effects was carried out using the GLM procedure in SPSS 13.0 for Windows. The fixed model was as follows: $Y_{ijkl}=u+m_{j}+s_{i}+t_{k}+\beta_{x}+e_{ijkl}$, where $Y_{ijkl}$ is daily milk yield, peak milk yield or persistence of genotype j in the ith year and season, u is the observed values' least square mean, $m_{j}$ is the effective value of genotype j, $s_{i}$ is the ith year and season's effective value, β is the somatic score's regression coefficient, x is somatic score and $e_{ijkl}$ is the random residual effect (Fang et al., 2014).

## 3 Results

### 3.1 The results of the pBI-CMV3-GPAM and pGPU6-shRNA-GPAM vector construction and transfection

**Figure 1:**
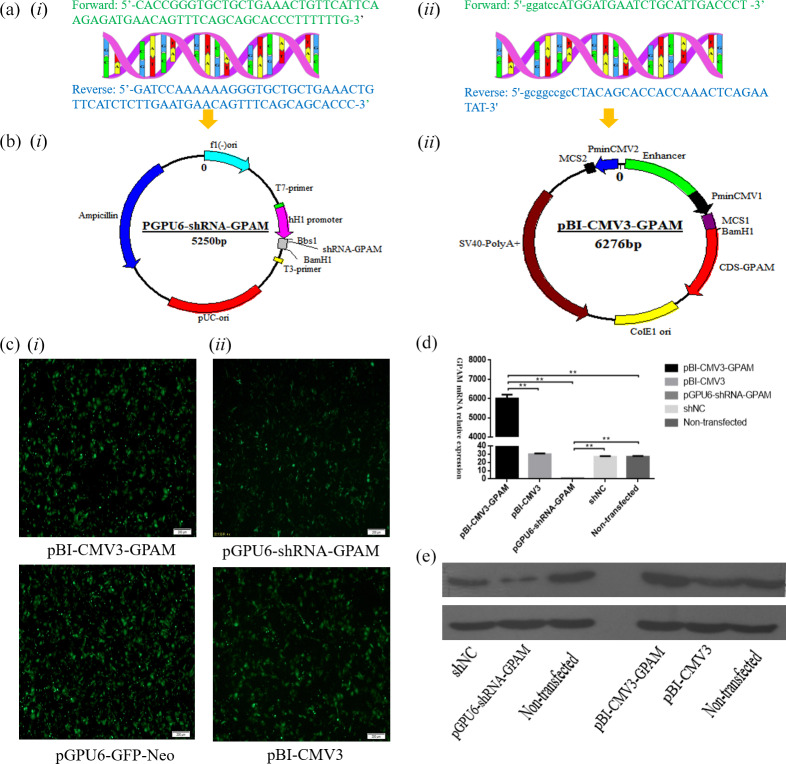
The expression of the *GPAM* gene effect on the synthesis of triglyceride in bMECs. **(a)** The bovine *GPAM* gene's primer sequences: **(i)** the bovine *GPAM* gene's shRNA primer sequences; **(ii)** primer sequence of *GPAM* gene's CDS region. **(b)** Construction of the pBI-CMV3-GPAM and pGPU6-shRNA-GPAM vector: **(i)** the bovine *GPAM* RNAi vector; **(ii)** the bovine *GPAM* gene overexpression vector. **(c)** Green fluorescence's expression was observed under a fluorescence microscope. **(d)** *GPAM* mRNA expression levels in bMECs. **(e)** *GPAM* protein expression levels in bMECs.

Sequence analysis showed that the shRNA, which targeted the *GPAM* gene's oligonucleotide sequence, was cloned and constructed into the Bbs I and BamH I sites of the pGPU6/GFP/Neo vector. Moreover, sequence analysis showed that the DNA fragments, including CDS sequences of *GPAM*, that were obtained by PCR and pBI-CMV3 plasmid were selected as the vector to overexpress *GPAM*. The cells in the pGPU6-shRNA-GPAM group and the pBI-CMV3-GPAM group were observed under fluorescent microscopes beginning 24 h after transfection for the expression of GFP as an indicator of successful transfection (Fig. 1c-i, ii).

### 3.2 The mRNA and protein expression levels of the *GPAM* gene in bMECs were overexpression and knockdown

The effect of *GPAM* was investigated through RNAi and overexpression of *GPAM*. The results of the analysis revealed that the expression levels of *GPAM* mRNA in the *GPAM* knockdown transfection group was substantially lower than that in the pGPU6-shRNA-NC group (Fig. 1d, p < 0.01), and the mRNA expression levels of *GPAM* in the pBI-CMV3-GPAM transfection group was considerably upregulated (Fig. 1d, p < 0.01).

Moreover, we also investigated the effects of *GPAM*-specific RNAi and overexpression on the mRNA expression of *GPAM* and on *GPAM*'s protein levels. Western blotting results showed that the pGPU6-shRNA-GPAM transfected bMECs' protein expression was downregulated in comparison with that of the negative control group cells (Fig. 1e, p < 0.01), whereas the pBI-CMV3-GPAM transfected bMECs' protein expression was upregulated in relation to that of the control group cells (Fig. 1e, p < 0.01). These western blotting results were consistent with the RT-qPCR results, indicating that the RNAi and overexpression of *GPAM* affect both the protein and mRNA levels of *GPAM*.

### 3.3 *GPAM* gene expression increases the intracellular triglyceride content in bMECs

The effect of pBI-CMV3-GPAM and pGPU6-shRNA-GPAM transfection on the triglyceride levels in bMECs was assessed and showed a substantial decrease in the triglyceride levels in the bMECs of the RNA interference group (p < 0.05, Table 2). The TG content, in comparison, increased considerably in bMECs due to the overexpression of the *GPAM* gene, indicating that *GPAM* plays a cardinal role in TG synthesis in the lipid metabolism pathway of bMECs.

**Table 2:** The TG and total protein contents of each transfected group in bMECs.

Sample	Triglyceride	Total protein
	content	content
	(mmol L${}^{-1}$)	(mg mL${}^{-1}$)
pGPU6-shRNA-GPAM	0.127 ± 0.027	4.82 ± 0.61
pBI-CMV3-GPAM	0.278 ± 0.022	4.39 ± 0.41
pBI-CMV3	0.175 ± 0.016	4.72 ± 0.31
pGPU6-shRNA-NC	0.181 ± 0.032	4.35 ± 0.24
Negative control	0.177 ± 0.019	4.23 ± 0.38

Note that values are expressed as the mean ± SE. pGPU6-shRNA-GPAM refers to cells transfected with the *GPAM* RNAi vector. pBI-CMV3-GPAM refers to cells transfected with the pBI-CMV3-GPAM vector. pGPU6-shRNA-NCC refers to cells transfected with the pGPU6-shRNA-NC vector. Negative Control refers to non-transfected cells.

### 3.4 Genetic diversity of SNPs in the *GPAM* gene in a Chinese Holstein cow population

**Figure 2:**
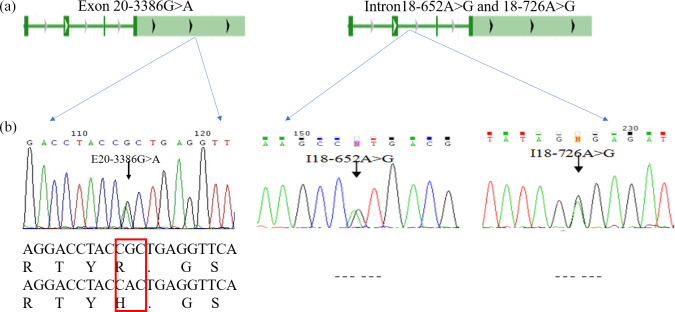
Analysis and sequencing of SNPs in the *GPAM* gene. Panel **(a)** shows the determination and sequence analysis of SNPs in the *GPAM* gene's functional regions; panel **(b)** shows that there was a G to A substitution at position 3386 bp of the 20th exon in the * GPAM* gene that replaced arginine with histidine, and there was no alteration in amino acids at locations I18-726A > G and I18-652A > G.

The PCR products were consistent and had good specificity with the target, which could be used for sequencing. Upon analysis of the sequencing result, a peak was obtained in the mutations of the three SNPs (E20-3386G > A, I18-652A > G and I18-726A > G; Fig. 2b). At location 3386 bp in the 20th exon, there was a G > A substitution that resulted in the replacement of arginine with histidine (Fig. 2b); there was also an A > G substitution at position 652 and 726 bp in the 18th intron. Allele A had a frequency of 0.26 and 0.44 at the I18-652 and I18-726 loci in the Holstein cow population respectively. At the E20-3386 polymorphism site, the dominant allele G had a frequency of 0.43 in the Chinese Holstein cows. The SNP's genotype distribution at E20-3386G > A, I18-652A > G and I18-726A > G in the studied cow population is shown in Table 3. The results showed that there were three polymorphisms in the intron and exon regions of the bovine *GPAM* gene.

**Table 3:** SNPs of the *GPAM* gene in China Holstein dairy cattle.

Type	Frequency				
	Allele frequency		Genotype frequency		
E20-3386G > A	G (0.70)	A (0.30)	GG (0.43)	GA (0.52)	AA (0.04)
I18-652A > G	A (0.49)	G (0.51)	AA (0.26)	GA (0.46)	GG (0.27)
I18-726A > G	A (0.65)	G (0.35)	AA (0.44)	GA (0.43)	GG (0.13)

### 3.5 Correlation analyses of *GPAM* polymorphisms with milk quality in China Holstein cows

The lactation traits of the Holstein cattle population include milk yield, milk fat percentage, milk protein percentage and somatic cell number. The mean value and standard error of each trait of the Holstein cattle population are shown in Table 4. The connections between *GPAM* gene polymorphisms and milk quality traits in Chinese Holstein cows were analysed. The results show that the three SNPs (E20-3386G > A, I18-652A > G and I18-726A > G) have substantial links with milk quality traits.

**Table 4:** The number of records and the means and standard deviations for the Chinese Holstein dairy cattle milk traits involved in the association analyses.

Traits	N	LSM	SE
Milk yield (kg)	241	27.3026	7.1601
Protein (%)	241	3.4790	0.2373
Lactose (%)	241	4.7179	0.1554
Dry matter (%)	241	13.8160	0.6408
SCC (10${}^{4}$ mL${}^{-1}$)	241	53.6768	49.9919
BUN (%)	241	22.5966	3.1689
FCM (kg)	241	35.7797	9.7822
Fat (%)	241	4.7300	0.3181
TFC (kg)	241	1.2914	0.1045

N refers to the number of samples, LSM is the least square mean, SE is the standard error, SCC is the somatic cell count, BUN is the urea nitrogen, FCM is the fat-corrected milk and TFC is the total fat content.

We found that the E20-3386G > A SNP of the *GPAM* gene was potentially correlated with fat, total fat content (TFC), protein and somatic cell count (SCC) (p < 0.05; Table 5). Individuals with the GA genotype possessed higher milk fat (5.03 ± 0.04) and TFC (1.38 ± 0.04 kg) than homozygotes with AA and GG in the studied population of Chinese Holstein cows (p < 0.05). Individuals cows with the GA genotype also had higher milk protein rate (3.54 ± 0.03) than AA genotype individuals (3.43 ± 0.08), and individuals with the AA genotype had a higher somatic cell count (110.92 ± 35.14) than GG genotype individuals (40.75 ± 6.11) and GA genotype individuals (58.18 ± 8.30). The I18-652A > G SNP was correlated with fat, protein, dry matter and somatic cell count (p < 0.05; Table 5). Individuals with the AA genotype had higher milk fat (4.89 ± 0.02) and TFC (1.40 ± 0.02 kg) than GA and GG homozygotes (p < 0.05). In terms of milk protein rate, values from GG genotype individuals (3.54 ± 0.03) and GA genotype individuals (3.48 ± 0.02) were significantly higher than that of AA genotype individuals (3.41 ± 0.03). In the context of dry matter content, values from GG genotype individuals (13.96 ± 0.09) were significantly higher than that of GA genotype individuals. The number of somatic cells in the GG genotype (56.78 ± 6.55) and GA genotype (60.37 ± 5.16) was significantly higher than that for the AA genotype (38.52 ± 4.04). The I18-726A > G SNP of the *GPAM* gene was significantly correlated with milk protein rate, milk yield, dry matter and somatic cell count (p < 0.05; Table 5), and this SNP may also have a significant correlation with the milk fat rate (p = 0.051). In terms of milk yield, the AA genotype (28.51 ± 0.71) was significantly higher than the GA genotype (26.22 ± 0.70) and the GG genotype (26.86 ± 1.17). The milk protein rate in the GA genotype (3.52 ± 0.02) was significantly higher than that for the AA genotype (3.42 ± 0.02). The dry matter content in the GG genotype (13.99 ± 0.11) was significantly higher than that of the AA genotype (13.72 ± 0.06). With respect to the somatic cell count, GG genotype individuals showed significantly higher values (66.31 ± 11.53) than AA genotype individuals (46.27 ± 4.00). Thus, the polymorphisms in *GPAM* could alter the milk quality traits attributed to the presence of a type of nucleotide.

**Table 5:** The association of the three SNPs in the *GPAM* gene with milk quality traits in Chinese Holstein dairy cattle.

Milk quality	E20-3386G > A genotype						I18-652A > G genotype						I18-726A > G genotype					
traits	GG (n=100)		GA (n=130)		AA (n=11)		AA (n=63)		GA (n=112)		GG (n=66)		AA (n=105)		GA (n=104)		GG (n=32)	
	LSM	SE	LSM	SE	LSM	SE	LSM	SE	LSM	SE	LSM	SE	LSM	SE	LSM	SE	LSM	SE
Milk yield (kg)	28.16	1.12	26.07	1.12	27.52	3.94	28.69	0.86	26.77	0.72	26.89	0.80	28.51${}^{a}$	0.71	26.22${}^{b}$	0.70	26.86${}^{\mathrm{ab}}$	1.17
Protein (%)	3.45${}^{a}$	0.03	3.54${}^{b}$	0.03	3.43${}^{\mathrm{ab}}$	0.08	3.41${}^{a}$	0.03	3.48${}^{b}$	0.02	3.54${}^{b}$	0.03	3.42${}^{a}$	0.02	3.52${}^{b}$	0.02	3.52${}^{b}$	0.04
Lactose (%)	4.74	0.02	4.70	0.02	4.72	0.08	4.73	0.02	4.71	0.02	4.72	0.02	4.73	0.01	4.70	0.01	4.72	0.03
Dry matter (%)	13.80	0.09	13.92	0.08	13.95	0.06	13.77${}^{\mathrm{ab}}$	0.08	13.76${}^{a}$	0.06	13.96${}^{b}$	0.09	13.72${}^{a}$	0.06	13.86${}^{\mathrm{ab}}$	0.06	13.99${}^{b}$	0.11
SCC (10${}^{4}$ mL${}^{-1}$)	40.75${}^{a}$	6.11	58.18${}^{a}$	8.30	110.92${}^{b}$	35.14	38.52${}^{a}$	4.04	60.37${}^{b}$	5.17	56.78${}^{b}$	6.55	46.27${}^{a}$	4.00	57.27${}^{\mathrm{ab}}$	5.12	66.31${}^{b}$	11.53
BUN (%)	22.46	0.38	22.44	0.44	24.20	2.03	22.27	0.38	22.78	0.31	22.60	0.38	22.35	0.31	22.96	0.32	22.25	0.51
FCM (kg)	35.42	1.24	33.36	1.40	38.34	6.75	35.04	1.13	35.73	1.03	36.56	1.06	35.16	0.88	36.22	1.06	36.37	1.59
Fat (%)	4.23${}^{a}$	0.07	4.45${}^{a}$	0.03	5.03${}^{b}$	0.04	4.89${}^{a}$	0.03	4.27${}^{a}$	0.04	4.19${}^{b}$	0.04	4.71	0.02	4.78	0.01	4.81	0.04
TFC (kg)	1.19${}^{a}$	0.05	1.16${}^{a}$	0.03	1.38${}^{b}$	0.04	1.40${}^{a}$	0.02	1.14${}^{b}$	0.03	1.13${}^{b}$	0.02	1.34	0.06	1.25	0.04	1.29	0.02

Note that significant differences (p < 0.05) among the mean values of the traits are shown using different lowercase letters (${}^{a}$, ${}^{b}$) in the same column. SE refers to standard error, LSM is the least square mean, FCM is the fat-correctedmilk, SCC is the somatic cell count, BUN is urea nitrogen and TFC is the total fat content.

## 4 Discussion

In the animal industry, fat traits are one of the most important characteristic with respect to commercial value. Many factors affect fat traits, including nutrition, management and genetics (Keogh et al., 2015; Sakamoto et al., 2014). In animal breeding and genetics, a number of techniques have been introduced for the analysis of molecular markers, including gene localisation, gene cloning, germplasm resource assessment, genetic mapping, the use of heterosis and molecular marker-assisted selection (Beuzen et al., 2000; de Los Campos et al., 2013; Thompson et al., 2015). The *GPAM* gene depends on physiological condition including nutrition and hormones (Harada et al., 2007; Onorato et al., 2005). The *GPAM* gene plays an important role in phospholipid and triglyceride synthesis (Lewin et al., 2004, 2005; Turnbull et al., 2001). Previous work on the *GPAM* gene has mostly focused on the function of the gene, whereas less research has been undertaken on polymorphisms of the *GPAM* gene. The expression levels of the *GPAM* gene were found to be increased during lactation in bovine (Bionaz et al., 2008), and the function of the *GPAM* gene in bovine is the same as mouse hepatic tissue (Wendel et al., 2013). The *GPAM* is located on BTA26, and the SNP of *GPAM* is linked with the fatty acid concentration (Stoop et al., 2009). To verify the role of the *GPAM* gene in triglyceride synthesis, experiments have been carried out on mice; this research suggested that the *GPAM* gene has various effects after knockout, including lighter gonadal fat pads, reduced weight, reduced plasma triglyceride levels and decreased liver triglyceride content. Thus, these results strongly suggest that the *GPAM* gene has a vital role in the synthesis of triglycerides in liver and plays a considerable role in the regulation of the fatty acid components of glycerophosphatides (Dawson et al., 2006; Jung et al., 2013). This result is consistent with our previous work (Yu et al., 2017), which found that the *GPAM* gene influences the TG content in bovine fetal fibroblasts (BFFs), the knockdown of the *GPAM* gene could cause the triglyceride in BFFs to significantly decrease and the overexpression of *GPAM* gene significantly increased the TG level compared with the BFF control group. This gives us more strong evidence that the *GPAM* gene has a role in fat metabolism and triglyceride synthesis (Yu et al., 2017).

As the relation between the *GPAM* gene and bovine lipid metabolism was confirmed in BFFs, we examine the knockdown and overexpression of *GPAM* in bMECs in the present study. In bMECs, the knockdown of the *GPAM* gene level of triglyceride was significantly decreased compared with the control group, whereas the overexpression of the *GPAM* gene significantly increased the TG level compared with the bMEC control group.

Moreover, previous work on mice has revealed that animals lacking the *GPAM* gene were much lighter, had a lower fat content in their gonads and had a 40 % lower TG content in their livers compared with wild mice (Wendel et al., 2013). In this study, the results showed that the TG content increased substantially with the overexpression of *GPAM* in bMECs; however when *GPAM* was knocked down in bMECs, the TG content decreased significantly

Three novel SNPs of the *GPAM* gene (E20-3386G > A, I18-652A > G and I18-726A > G) were also found to be significantly correlated with fat, the total fat content (TFC), protein, milk yield, dry matter and somatic cell count (SCC) in this study. Our data suggest that *GPAM* polymorphism may be one of the significant genetic factors affecting milk quality; therefore, it may provide a competent marker for milk and meat quality traits in dairy cattle breeding and production. It could also be used to develop or improve the dual purpose of cattle breeds.

## 5 Conclusions

In summary, TG synthesis can be promoted by the *GPAM* gene in the fat metabolism pathway of bMECs. Furthermore, milk quality can be significantly affected by mutations in the *GPAM* gene's functional domain. *GPAM* plays a pivotal role in the regulation of phospholipid levels and cellular triglyceride, and its mutations could serve as a competent molecular marker that could be utilised for marker-assisted selection.

## Data Availability

The data sets are available upon request from the corresponding author.
